# An unusual complication of Mitek suture anchor use in primary treatment of flexor digitorum profundus tendon laceration: a case report

**DOI:** 10.1186/1757-1626-2-9319

**Published:** 2009-12-14

**Authors:** Dimitrios Giannikas, Efstratios Athanaselis, Charalambos Matzaroglou, Alkis Saridis, Minos Tyllianakis

**Affiliations:** 1 Department of Orthopaedic Surgery, University Hospital of Patras, Greece

## Abstract

**Introduction:**

A case of an osteolysis by Mitek anchor-suture is presented.

**Case presentation:**

A case of index finger's flexor digitorum profundus tendon primary reconstruction with the use of Mitek anchor is presented here. Within a 14 month period, Mitek suture anchor caused local foreign body reaction with osteolysis and ulceration of the palmar skin of the finger while on the other hand tendon's healing was successfully completed.

**Conclusion:**

Mitek anchor-sutures can cause an aseptic inflammatory reaction which represents a typical biologic response to a foreign body. Concomitant osteolysis can drive to loosening and migration of the implant.

## Introduction

Mitek anchors are frequently used in treatment of flexor and extensor tendons' laceration and avulsion. In our case the use of such an anchor resulted in flexor tendon healing despite the fact that it was complicated by a foreign body reaction (granulomatosis and osteolysis). These findings led to Mitek anchor removal 14 months after the implantation.

## Case presentation

A 25-year-old Greek, industrial machine operator was admitted to our clinic after a traumatic laceration of FDP tendon of the index finger, distally to the fourth cruciate pulley of the right hand.

Clinical examination revealed a transverse wound of approximately 1 cm on the palmar side of the index finger just proximally to the DIP joint with associate inability of flexion of the distal phalange. Under local anesthesia, wound was extended distally and mainly proximally in order to expose the ends of the tendon. The use of a non-absorbable Mitek suture anchor was decided in order to reattach the tendon. After drilling a hole on the palmar side of the base of third digit, a mini Mitek anchor was introduced. Adequate anchoring of the Mitek was checked and the tendon was firmly fixed with the remaining distal stump. The hand was immobilized in a boxing glove and a 2^nd ^generation cephalosporin was administrated to the patient for a week.

Wound was checked and changed every two days and sutures were removed 15 days after. Meanwhile wound healing had been completed without complications. Passive exercises of finger flexion were advised via a Kleinert splint for six weeks, followed by active assisted exercises for a further period of two months.

**Figure 1:**
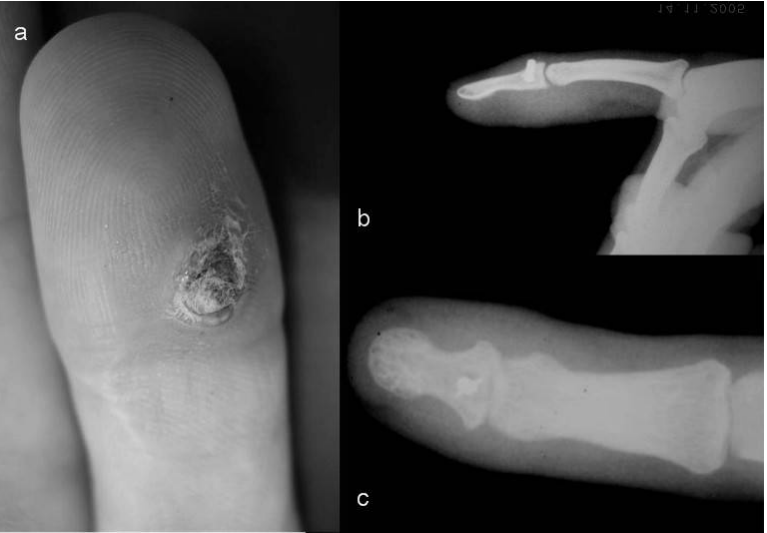
**(a) recurrent ulcer on the palmar side of distal phalange of index finger, (b) lateral and (c) anterioposterior x-rays**.

Patient attended our clinic 14 months later complaining of a recurrent ulcer on the palmar side of the distal phalange (Figure 1a) of the finger during the last two months. A palmar subcutaneous mass had appeared two months before on the palmar side of distal third of the index finger. The mass was slightly tender on palpation and kept growing until becoming an ulcer for which, antibiotic therapy suggested by another doctor, was received periodically.

**Figure 2:**
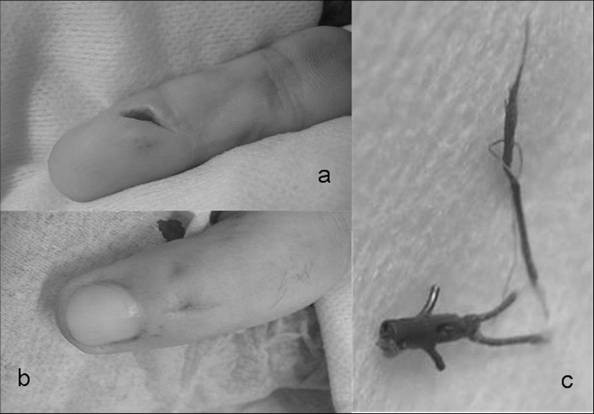
**(a) palmar and (b) dorsal approach to distal digit and (c) the removed Mitek suture anchor**.

On the X-ray control dorsal migration of the Mitek anchor and osteolysis of the surrounding bone were revealed (Figure 1b and 1c). Materials (anchor and non-absorbable sutures) were removed operatively by dorsal and palmar approach (Figure 2a and 2b), pushing the anchor forward in order to avoid pull-out resistance (Figure 2c). Tendon's healing was found completed and functional. Bacteriological cultures were taken from the ulcer which was excised and the palmar incision was primaly sutured. The dorsal incision was left open for drainage. Finally no pathogenic organism was isolated from the wound cultures. The wound was closed uneventfully. No recurrence was noticed.

## Discussion

According to Leddy and Packer classification, injuries of distal end of FDP tendons may be avulsions from its attachment at the base of the distal phalanx without a concomitant avulsion fracture (type 1), avulsions with a small avulsion fragment from the volar side of the distal phalanx' base (type 2) and avulsions involving a large bony fragment from the same side (type 3) [1]. Very distal FDP lacerations of zone I with distal tendon stump insufficient to allow for a satisfactory core suture, are basically the same as avulsion injuries of type 1 and treatment technique is also the same.

A widely known and accepted method of treating such lesions is a pullout suture tied over a button on the nail plate. The need of suture removal drove to the development of a variety of suture techniques with an unlocked core suture [2,3]. However, the external dorsal button is often a source of inconvenience for the patient with potential risks associated with nail plate deformities, nail fold necrosis and infections tracking along the sutures. Small suture anchors provide a satisfactory alternative.

Absorbable and non-absorbable implants as suture anchors used in fracture management (and in knee and shoulder surgery) can cause osteolysis of the surrounding bone [4-6]. The exact cause of the tunnel-widening seen on radiographs is not known entirely, but in some instances, it has been associated with a foreign-body reaction and loosening of the implant [7]. Migration of anchors has been described among complications in the use of these materials and osteolysis could be a cause as it drives to loosening of anchor and bone interface [8]. On the other hand, these materials can cause an aseptic inflammatory reaction which represents a typical biologic response to a foreign body.

There are few reported cases of infections after surgical implantation of suture anchors and we can assume that the risk of postoperative infection is not high. However, infection can occur after these procedures, and therefore must be always in mind.

In our case anchor had been inserted in the palmar surface of distal phalanx at approx. 45° in a retrograde way as it is recommended, for better resistance against to tendon's pull-out forces. This manner of insertion decreases the risk of mechanically caused loosening and as patient's finger was asymptomatic macroscopically for about eleven months, septic loosening can be excluded. Granulomatosis caused by a biological foreign body reaction to used materials (titanium of anchor, Ethibond sutures or both) drove to ulcer creation and this aseptic inflammation is responsible for surrounding osteolysis.

Despite the inflammatory response at the area, the procedure of tendon healing was successfully completed and the materials could be removed without other consequences.

## Conclusion

Mitek anchor is an effective treatment choice in flexor and extensor tendon laceration and avulsion. However, as any foreign material, can induce a foreign body reaction causing granulomatosis and surrounding osteolysis. This is indeed an unusual complication not related to infection but foreign body reactions may occur even with biocompatible materials. Granulomatous reaction to a Mitek anchor used in a confined area such as a digit resulted in an unexpected complication which fortunately did not affect tendon healing and soft tissue problems were solved by implant removal but.

## Consent

Written informed consent was obtained from the patient for publication of this case report and accompanying images. A copy of the written consent is available for review by the Editor-in-Chief of this journal.

## Competing interests

The authors declare that they have no competing interests.

## Authors' contributions

DG operated on the patient and supervised writing of the case report. EA wrote this article, contributed in the all concept, methodology of the essay reference gathering, and took part in the operation. CM and AS assisted in formulation of the text and MT supervised the writing of the discussion and complete the text enrichment. All authors read and approved the final manuscript.
